# Neurophysiological consequences of synapse loss in progressive supranuclear palsy

**DOI:** 10.1093/brain/awac471

**Published:** 2022-12-14

**Authors:** Natalie E Adams, Amirhossein Jafarian, Alistair Perry, Matthew A Rouse, Alexander D Shaw, Alexander G Murley, Thomas E Cope, W Richard Bevan-Jones, Luca Passamonti, Duncan Street, Negin Holland, David Nesbitt, Laura E Hughes, Karl J Friston, James B Rowe

**Affiliations:** Department of Clinical Neurosciences and Cambridge University Hospitals NHS Trust, University of Cambridge, Cambridge CB2 0SZ, UK; MRC Cognition and Brain Sciences Unit, University of Cambridge, Cambridge CB2 7EF, UK; Department of Clinical Neurosciences and Cambridge University Hospitals NHS Trust, University of Cambridge, Cambridge CB2 0SZ, UK; Department of Clinical Neurosciences and Cambridge University Hospitals NHS Trust, University of Cambridge, Cambridge CB2 0SZ, UK; MRC Cognition and Brain Sciences Unit, University of Cambridge, Cambridge CB2 7EF, UK; Department of Clinical Neurosciences and Cambridge University Hospitals NHS Trust, University of Cambridge, Cambridge CB2 0SZ, UK; MRC Cognition and Brain Sciences Unit, University of Cambridge, Cambridge CB2 7EF, UK; Washington Singer Laboratories, University of Exeter, Exeter EX4 4QG, UK; Department of Clinical Neurosciences and Cambridge University Hospitals NHS Trust, University of Cambridge, Cambridge CB2 0SZ, UK; Department of Clinical Neurosciences and Cambridge University Hospitals NHS Trust, University of Cambridge, Cambridge CB2 0SZ, UK; MRC Cognition and Brain Sciences Unit, University of Cambridge, Cambridge CB2 7EF, UK; Department of Clinical Neurosciences and Cambridge University Hospitals NHS Trust, University of Cambridge, Cambridge CB2 0SZ, UK; Department of Clinical Neurosciences and Cambridge University Hospitals NHS Trust, University of Cambridge, Cambridge CB2 0SZ, UK; MRC Cognition and Brain Sciences Unit, University of Cambridge, Cambridge CB2 7EF, UK; Department of Clinical Neurosciences and Cambridge University Hospitals NHS Trust, University of Cambridge, Cambridge CB2 0SZ, UK; Department of Clinical Neurosciences and Cambridge University Hospitals NHS Trust, University of Cambridge, Cambridge CB2 0SZ, UK; Department of Clinical Neurosciences and Cambridge University Hospitals NHS Trust, University of Cambridge, Cambridge CB2 0SZ, UK; Department of Clinical Neurosciences and Cambridge University Hospitals NHS Trust, University of Cambridge, Cambridge CB2 0SZ, UK; MRC Cognition and Brain Sciences Unit, University of Cambridge, Cambridge CB2 7EF, UK; Wellcome Centre for Human Neuroimaging, University College London, London WC1N 3AR, UK; Department of Clinical Neurosciences and Cambridge University Hospitals NHS Trust, University of Cambridge, Cambridge CB2 0SZ, UK; MRC Cognition and Brain Sciences Unit, University of Cambridge, Cambridge CB2 7EF, UK

**Keywords:** progressive supranuclear palsy, MEG, modelling, PET, SV2A

## Abstract

Synaptic loss occurs early in many neurodegenerative diseases and contributes to cognitive impairment even in the absence of gross atrophy. Currently, for human disease there are few formal models to explain how cortical networks underlying cognition are affected by synaptic loss. We advocate that biophysical models of neurophysiology offer both a bridge from preclinical to clinical models of pathology and quantitative assays for experimental medicine. Such biophysical models can also disclose hidden neuronal dynamics generating neurophysiological observations such as EEG and magnetoencephalography. Here, we augment a biophysically informed mesoscale model of human cortical function by inclusion of synaptic density estimates as captured by ^11^C-UCB-J PET, and provide insights into how regional synapse loss affects neurophysiology. We use the primary tauopathy of progressive supranuclear palsy (Richardson’s syndrome) as an exemplar condition, with high clinicopathological correlations. Progressive supranuclear palsy causes a marked change in cortical neurophysiology in the presence of mild cortical atrophy and is associated with a decline in cognitive functions associated with the frontal lobe. Using parametric empirical Bayesian inversion of a conductance-based canonical microcircuit model of magnetoencephalography data, we show that the inclusion of regional synaptic density—as a subject-specific prior on laminar-specific neuronal populations—markedly increases model evidence. Specifically, model comparison suggests that a reduction in synaptic density in inferior frontal cortex affects superficial and granular layer glutamatergic excitation. This predicted individual differences in behaviour, demonstrating the link between synaptic loss, neurophysiology and cognitive deficits. The method we demonstrate is not restricted to progressive supranuclear palsy or the effects of synaptic loss: such pathology-enriched dynamic causal models can be used to assess the mechanisms of other neurological disorders, with diverse non-invasive measures of pathology, and is suitable to test the effects of experimental pharmacology.

## Teaser

Adams *et al.* use measures of synaptic density from PET to enhance biophysical models of cortical dynamics in neurodegeneration and reveal how synaptopathy affects local activity. The network properties predict individual differences in behaviour, providing a new way to link synaptic loss, neurophysiology and cognitive change.

## Introduction

Human neurodegenerative diseases are heterogeneous in their symptoms, progression and molecular biology, but they all call for mechanistic explanations of the pathophysiology underlying cognitive impairment.^1–4^ This may be met by biophysically informed models of brain-network dynamics that integrate patient-specific measures of neuropathology. We propose that by embedding neuropathological information in individualized disease models, one could establish bridges between preclinical and clinical models of disease, facilitate experimental medicine and inform precision medicine. We therefore sought to enrich biophysically informed generative models of cortical neurophysiology, inverted from magnetoencephalography (MEG), with markers of neuropathological severity from PET.

We focus on synapse loss as the neuropathology feature, which is common across many neurodegenerative diseases and closely related to the severity of dementia.^5–12^ This kind of synapse loss is a consequence of protein misfolding, aggregation and inflammation in multiple disorders, and begins before cell death.^13^ Post-mortem studies have identified cell- and region-specific changes in synaptic density.^14–16^ Quantification of region-specific synaptic density is now possible *in vivo* with PET, using ligands for the synaptic vesicle protein 2A.^12,17,18^ However, less is known about the impact of this synaptic loss on the neurophysiological function of local cortical networks.^7^

To characterize the relationship between synaptic loss and cortical neurophysiology, we use the primary tauopathy of progressive supranuclear palsy (PSP) as an exemplar condition. Within the group of frontotemporal lobar degeneration pathologies, PSP has very high clinicopathological correlation. Over and above the motor impairments of PSP, it is associated with marked decline in cognitive function and physiological responses, especially cognitive functions associated with the frontal lobe.^19–21^ These frontal physiological and cognitive changes occur in Richardson’s syndrome as well as the PSP-Frontal phenotype, despite only mild cortical atrophy.^22^ The discrepancy between severe functional deficits and mild atrophy has been proposed to result from changes in synaptic density and loss of major neurotransmitter systems in the frontal lobe.^22–27^ PSP synaptic loss is severe in multiple cortical regions at post-mortem and *in vivo*,^10,28^ making the disorder ideally suited to demonstrate the relationship between synaptic loss and cortical function.

We had three principal aims. First, to develop a method for pathology-enriched dynamic causal modelling (DCM), combining MEG with PET data. Using this method, we could test for a relationship between synaptic density and inferred synaptic efficacy within the generators of MEG signals. Second, we sought to identify the subject-, layer- and cell-specific parameters that are most sensitive to changes in synaptic density. We focus on the synaptic loss and neurophysiological function of the frontal lobe (specifically inferior frontal gyrus) because of the cognitive profile of PSP. The third aim was to test the hypothesis that the neuronal parameter estimates are correlated with cognitive function. To achieve this, we undertook patient-based group-wise analyses using covariates that represent individual differences in synaptic density, clinical severity and neurophysiological response.

In pursuing these aims, we also considered the validity of the modelling and data analysis: face validity was established in terms of the accuracy of the generative model when explaining observed MEG (i.e. the model could reproduce realistic neurophysiological responses) in addition to the previous anatomical and neurophysiological studies on which the cortical microcircuit model is based.^29,30^ Construct validity, with generalization over comparable data, was assessed through analysis of reliability using the intraclass correlation coefficient (ICC) and a split half procedure (i.e. odd and even trials). Predictive validity was addressed using measures of disease severity and pathophysiology, and the associated laminar-specific synaptic disruption.^14–16^

## Materials and methods

### Participants

Eleven people with probable PSP Richardson’s syndrome^31^ underwent structural MRI, ^11^C-UCB-J PET and MEG. Whereas prominent presenting features can be cognitive and behavioural (e.g. in PSP-Frontal phenotype), all had progressed to Richardson’s syndrome by the time of the study. Participants were recruited from the Cambridge Centre for Parkinson-plus and gave written informed consent in accordance with the Declaration of Helsinki (1991). Their clinical and cognitive assessment included the Mini Mental State Examination, revised Addenbrookes Cognitive Examination, Cambridge Behavioural Inventory, Hayling sentence completion test, INECO Frontal Screening, Progressive Supranuclear Palsy Rating Scale, Frontal Assessment Battery (FAB) and Graded Naming Test. Demographic and clinical data of participants are summarized in Supplementary Table 1.

### Neuroimaging data acquisition

During MEG, participants were exposed to a roving auditory oddball stimulus train, as described in Adams *et al*.^19^ MEG/electrophysiological data were recorded at 1000 Hz using a 306-channel Vectorview acquisition MEG system (Elekta Neuromag) located in an Elekta Neuromag magnetically shielded room. Sensors are in triplets, as a pair of gradiometers and a magnetometer. Electrooculograms tracked eye movements vertically and horizontally, and five head-position indicator coils tracked the head position (500 Hz). EEG was simultaneously recorded using a 70 channel, EMG-compatible, EEG cap (Easycap). A 3D digitizer (Fastrak Polhemus Inc) recorded >100 scalp points, nasion and bilateral pre-auricular fiducial points.

For coregistration with the MEG data, T_1_-weighted structural MRI was collected in a 7 T Siemens TERRA scanner (magnetization-prepared two rapid gradient-echo sequence, echo time = 1.99 ms, repetition time = 4300 ms, 0.75 mm isotropic voxels). For one subject, the scan was collected in a 3 T Siemens PRIMSA scanner [with magnetization-prepared rapid gradient-echo (echo time = 2.9 ms, repetition time = 2000 ms, 1.1 mm isotropic voxels)] at the Wolfson Brain Imaging Centre, University of Cambridge.

Participants underwent a ^11^C-UCB-J PET scan, on a GE SIGNA PET/MR (GE Healthcare), with 90 min of dynamic imaging following ^11^C-UCB-J injection, and then attenuation correction including the use of a multi-subject atlas method^32^ and improvements to the brain coil component. Full details of the post-processing are provided in Holland *et al*.^10^ In brief, the data were attenuation corrected and aligned to a simultaneous subject-specific T_1_-weighted magnetic resonance image (echo time = 9.2 ms, repetition time = 3.6 ms, 1.0 mm isotropic voxels, 192 sagittal slices, in-plane voxel dimensions 0.55 × 0.55 mm subsequently interpolated to 1.0 × 1.0 mm; slice thickness 1.0 mm).

Regions were specified using the Hammersmith Atlas. Regional time–activity curves were extracted following the application of geometric transfer matrix partial volume correction to each dynamic image. Regions of interest were multiplied by a binary grey matter mask (>50% on the SPM12 grey matter probability map smoothed to PET spatial resolution). The non-displaceable binding potential of ^11^C-UCB-J was estimated as the measure of synaptic density, using the simplified reference tissue model with the centrum semiovale as the reference region (corrected for CSF and grey matter partial volume). Only the right inferior frontal gyrus data were carried over to this study.

### Data preprocessing

MEG data were acquired using a standard (roving) auditory mismatch negativity paradigm. The data were MaxFiltered (v.2.2, Elekta Neuromag) to remove external noise, correct for head motion and interpolate bad channels. Subsequent data processing was performed with the Statistical Parametric Mapping toolbox (SPM12 v.7771, Wellcome Trust Centre for Neuroimaging, UK) FieldTrip (fieldtriptoolbox.org) and Oxford Centre for Human Brain Activity Software Library (https://github.com/OHBA-analysis/osl-core) software in MATLAB (2019a, MathWorks, Natick, MA, USA). Data were downsampled to 500 Hz, band-pass filtered between 0.1 and 125 Hz and notched between 45–55 and 95–105 Hz. Bad channels were removed using osl_detect_artefacts, before independent component analysis was used to remove eye-motion artefacts. Data were then epoched from −100 to 400 ms relative to stimulus onset. Further artefact rejection used thresholding of MEG channels to remove bad trials (osl_detect_artefacts). The deviant and standard trials—that constitute the roving mismatch paradigm—were taken as the first and sixth trials of each stimulus train, respectively, following a change in auditory tone.

Conventional source reconstruction was performed using the coherence method (i.e. ‘COH’ option in SPM12), using subject-specific structural images. Source data time series were obtained using a region of interest—with a radius of 7 mm—for the reconstruction of regional responses. A single (representative) source was selected for subsequent analysis of between-subject differences: namely, the right inferior frontal gyrus, with the Montreal Neurological Institute template coordinate of [46, 20, 8]. This is a prefrontal source in the auditory hierarchy (of five sources) that generate the auditory evoked responses (and accompanying differences that constitute the mismatch negativity).

### Dynamic causal modelling

**Figure 1:**
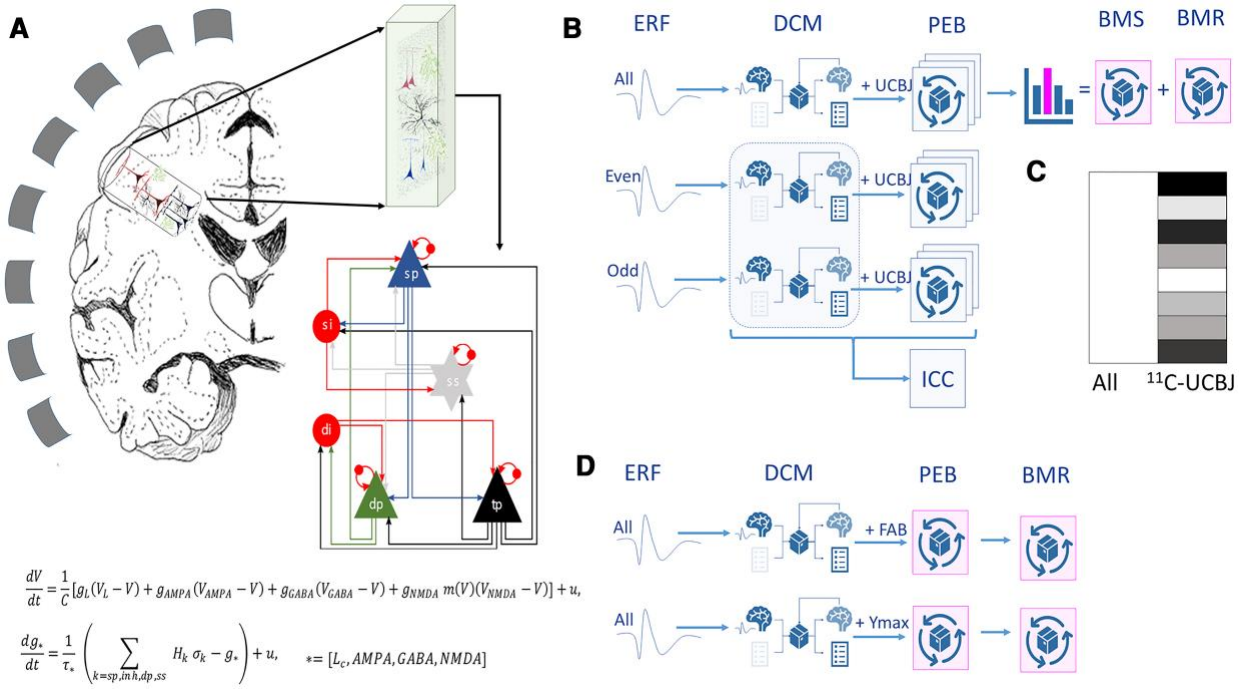
**Overview of the analysis**. (**A**) MEG detects the extra-cranial magnetic fields arising from cortical generators, via an invertible geometric lead field. The cortical region has a regularized structure of layers, with superficial (sp) and deep pyramidal cells (dp, tp), stellate cells in layer 4 (ss) and inhibitory interneurons in superficial (si) and deep (di) layers. The equations describe neuronal dynamics in terms of membrane potentials, V, and conductances of ion channels g, membrane capacitance, C, and endogenous fluctuations, *u*, with a passive leak current, *cL.* The conductances associated with each ion channels/receptors have time constants *τ*, while *σ_k_* refers to the non-negative afferent presynaptic firing from population *k*, scaled by afferent intrinsic connectivity. From generic priors, these parameters are optimized during model estimation. (**B**) Preprocessed MEG data from each participant were analysed at the first level using parallel DCM for all trials, and separately for odd trials and even trials. The odd and even trial analyses enabled the estimation of reliability (as ICC). For each dataset, first-level results were taken to group-wise parametric empirical Bayes (PEB) analysis, for which the design matrix includes a group average term and the synaptic density (from ^11^C-UCB-J PET) as empirical priors for each participant (**C**). Sixty-three such PEB models were run, one for every combination of synaptic parameters under the potential influence of individual differences in synaptic density. Bayesian model selection (BMS) and Bayesian model reduction (BMR) were used to identify the connections that are most likely to be related to synaptic loss. (**D**) Additional DCMs were run using PEB analyses that incorporated clinical scores (FAB) or peak evoked mismatch response (*Y*_max_) as empirical priors. ERF = event-related field.

Conductance-based DCM with six neuronal populations or cell types was used, based on the CMC-NMDA model described in Adams *et al*.^29^ (Fig. 1A). This neural mass mean-field model represents a local region of human cortex in terms of six cell populations: granular layer stellate cells, superficial layer pyramidal cells and both regular- and burst-firing pyramidal cells in deep cortical layers, plus a population of inhibitory neurons in both superficial and deep cortical layers. This extends the default four-population model in SPM12. The local field potential generated by this network is a weighted sum of each of the contributory cell populations. Neuronal responses are expressed in terms of the rates of change of membrane potentials and conductances, for each cell population according to the presence and time constants of AMPA, NMDA and GABA-mediated intrinsic (i.e. inter-and intra-laminar) coupling (Fig. 1A). The right inferior frontal gyrus event-related field time series for each participant was used for model inversion using standard (variational Laplace) procedures with a maximum of 64 iterations of the inversion scheme. Model parameters were optimized using the variational free energy bound on log model evidence (i.e. accuracy minus complexity). A list of the mean and variance (i.e. inverse precision) of prior model parameters is provided in Supplementary Table 2. These parameters pertain to synaptic time constants and rate constants that parameterize the efficacy of intrinsic (i.e. within source) connections among the six populations, which are assigned to cortical lamina.

The subject-specific parameter estimates (their mean and precision) were entered into parametric empirical Bayes (PEB) analyses (Fig. 1).^33^ These between-subject analyses were used to test the hypothesis that one or more synaptic parameters could be explained by individual differences in (PET derived) synaptic density measures from the inferior frontal gyrus region of interest. The ensuing PEB models were created from six sets of synaptic parameters: superficial and deep AMPA, NMDA and GABA. These 2 × 3 = 6 groups form a model space of 63 models (as 2^6^ − 1), where each model corresponds to a particular combination of synaptic parameters that could be influenced by synaptic density (Fig. 3A). The evidence (also known as the marginal likelihood) for each model was evaluated using the variational free energy (also known as an evidence lower bound). The resulting differential free energies of the ensuing PEB models were converted to posterior probabilities of each model, via the softmax operator. In this work, we focused on the synaptic parameters mediating responses to both standard and deviant stimuli, where the differential responses (that underlie the mismatch negativity) were modelled with parameters, mediating condition-specific changes in connectivity. This allowed us to use the amplitude in the mismatch negativity window as an independent marker of disease severity.

Further PEB analyses were undertaken (Fig. 1D) to assess the contributions of (i) the cognitive deficits as measured by the FAB, chosen because of its clinical utility, sensitivity to the presence of PSP and association with frontal lobe pathology; and (ii) a simple marker of the evoked physiological response, quantified here as the single maximal deflection during the mismatch window (*Y*_max_, 130–180 ms). Since DCM inverts from the physiological recordings, the question of independence of *Y*_max_ and DCM’s posterior parameters needs consideration. We note that (i) the DCM inversion uses the full time series of responses to standard and deviant tones over the trial window 0–500 ms; and (ii) it does not invert from the mismatch negativity time series, or invert from the *Y*_max_ of the differential evoked response. These PEB analyses looked for influences on the previous (six) sets of superficial and deep AMPA, NMDA and GABA connections.

Finally, these analyses were re-run for odd and even trials separately to create independent datasets and subsequent model inversion. The ICC (two-way random ICC class 2, assuming a consistent sample of raters^34^) was used as a measure of the within-subject reliability, using the odd and even trials’ data.

### Data availability

The MEG data preprocessing pipeline is available at https://github.com/AlistairPerry/FTLDMEGMEM. The DCM used in this study was adapted from https://gitlab.com/tallie/edcm, as described in Adams *et al*.^29^ with prior model parameters altered according to Supplementary Table 2. The data that support the findings of this study will be available from the corresponding author, upon reasonable request for academic (non-commercial) purposes, subject to restrictions required to preserve participant confidentiality. A data transfer agreement may be required.

## Results

**Figure 2:**
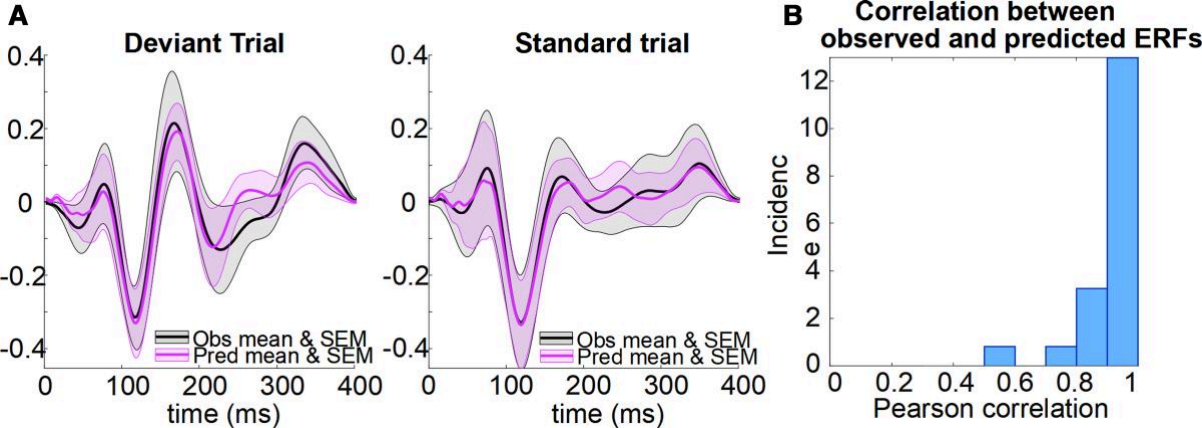
**The accuracy of the generative model of the evoked response**. (**A**) Event-related fields (ERFs) for the standard and deviant trials shown as a mean with SEM over all subjects. Observed data are in black and data predicted by the canonical microcircuit model are in purple. (**B**) The histogram illustrates the high correlations between observed and predicted ERFs for each participant.

The observed event-related fields and model event-related field predictions (Fig. 2A) were highly correlated (Fig. 2B, mean Pearson’s correlation = 0.86 ± 0.15), reflecting the validity of the generative models instantiated by the DCM.

**Figure 3:**
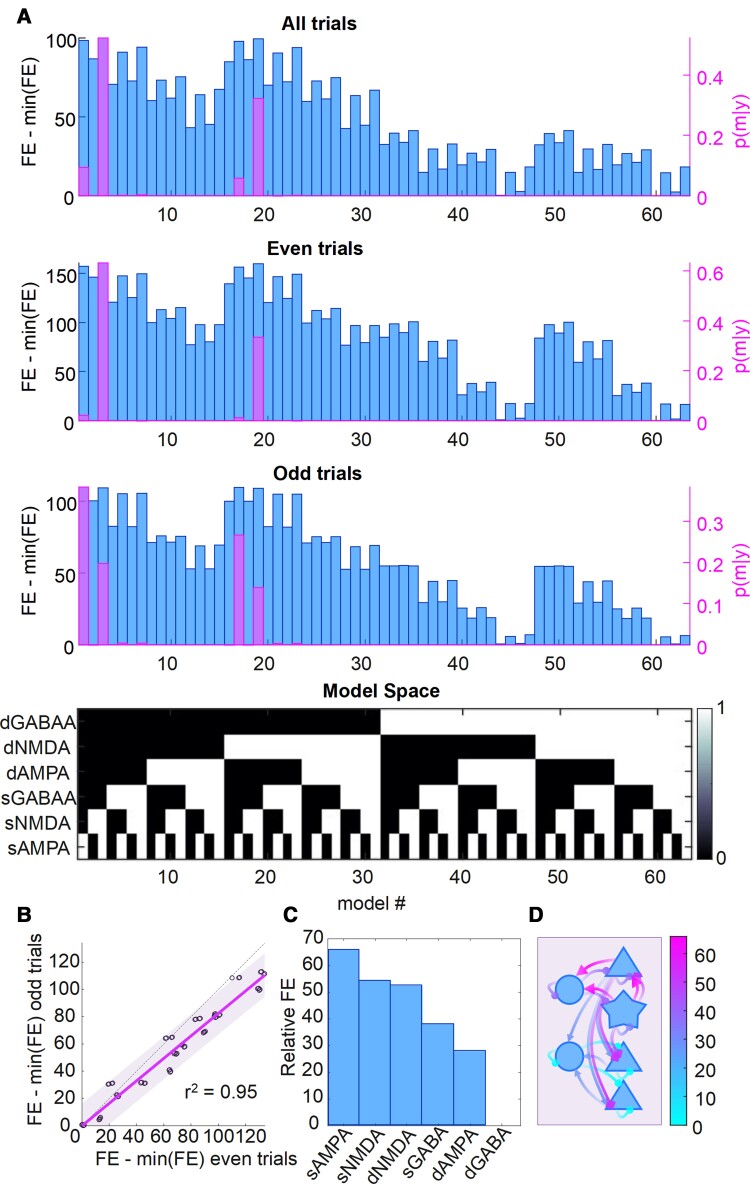
**The reliability of the free energy estimation over independent data per subject and over subjects**. (**A**) The free energy (FE, blue bars) for all 63 models for all trials (*top*), even trials (*middle*) and odd trials (*bottom*). The *right y*-axis (and pink bars) shows the posterior probability of each model. The model space, aligned with the bar charts above, is shown as the black and white matrix below (see Fig. 3 in Zeidman *et al.*^33^). (**B**) A comparison of the free energy for the most likely model plotted separately for even trials and odd trials for each subject, indicating the high reliability of the free energy estimate of the bound on (log)-model evidence. (**C**) The relative free energy for each connection group when considered in isolation. (**D**) The relative free energy for each connection group, with arrows distinguishing synapse type (triangle = AMPA; circle = GABA; diamond = NMDA). The scale for the colour map refers to the range of relative free energies across the groups.

To identify the relationship between each participant’s regional synaptic density and the estimates of synaptic efficacy, PEB was used to search for the best mapping from subject-specific synaptic density in right inferior frontal gyrus to different combinations of synaptic parameters in the DCM of the same region. These between-subject models were compared using their free energy (Fig. 3A, with the model space described in the lower matrix). The model space covers all combinations of superficial and deep AMPA, NMDA and GABA connections. The winning model of all available data (Fig. 3A, top) was the model in which superficial AMPA and NMDA synaptic connections were sensitive to PET measures of synaptic density (posterior probability = 0.52), followed closely by a model that also included deep NMDA connections (posterior probability = 0.32).

To assess the reliability of the PEB analyses, DCM was applied separately for odd and even trials. Results for ‘all’, ‘odd’ and ‘even’ trials are reported in Fig. 3A. The free energies of the models for ‘odd’ and ‘even’ trials were highly correlated over models, and over subjects for the most likely model (*r*^2^ = 0.95; Fig. 3B). The overall winning model was identical for the ‘all’ and ‘even’ conditions but differed slightly for the ‘odd’ trials’ data. However, this difference shows a related family nesting of synaptic groups. To quantify the relative importance of the nested model features, each set can be viewed in isolation in terms of its relative free energy (using all the available data) in Fig. 3C. The schematic in Fig. 3D illustrates the layout of these connections, coloured according to their relative free energy. Here, superficial AMPA connections rank the highest (i.e. most likely as a group to be associated with increased model evidence when informed by empirical ^11^C-UCB-J PET priors), followed in order by superficial NMDA connections, deep NMDA, superficial GABA, deep AMPA and finally deep GABA.

**Figure 4:**
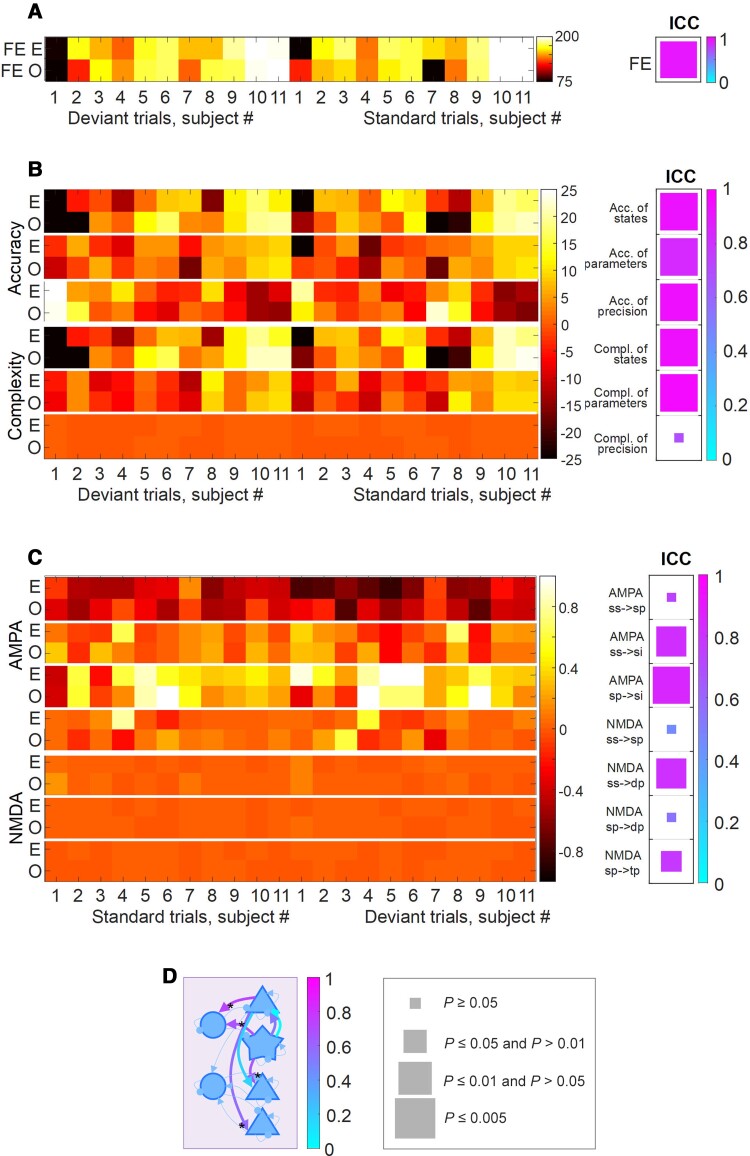
**The reliability of free energy, accuracy and complexity estimation**. (**A**) The free energy (FE) estimates for each subject shown separately by odd (O) and even (E) trials are shown with the ‘hot’ colour map on the left and the ICC reported with the ‘cool’ colour map on the *right*. For the ICC results, the size of the square relates to its frequentist significance level with the key provided at the *bottom*. (**B**) As before for the three accuracy and complexity measures, for the full model. (**C**) As before for the connection set found most likely to be correlated with synaptic density. (**D**) A schematic illustrating the reliability of neuronal parameter estimates with arrows coloured according to ICC(2) value using the ‘cool’ colour map. The asterisks denote the reliable connections.

The ICC was used to assess the reliability of the free energy estimates of the log evidence (Fig. 4A), using ‘odd’ and ‘even’ trials. The reliability was high, in terms of free energy of the full model (in which PET priors act over all layers and all receptor types), with ICC = 0.83 (*P* < 0.0001). We then tested separately the reliability of the accuracy and complexity that constitute the free energy (where log evidence equals accuracy minus complexity). The accuracy of the states, parameters and the precision and complexity of the states and parameters were again highly reliable (mean ICC = 0.85 ± 0.09, *P* < 0.005; Fig. 4B). However, the complexity of precision was not reliable (ICC = 0.43, *P* > 0.05).

Having confirmed reliability of model evidence estimates, ICCs were evaluated for the AMPA and NMDA synaptic parameter estimates that had shown the most evidence of the influence of individual differences in regional synaptic density (Fig. 4C). Assessing the reliability of single parameter estimates is not the most efficient way to assess reliability, due to conditional dependencies among the parameter estimates. Nonetheless, for four out of seven connections (superficial cortical layer AMPA, superficial cortical layer NMDA and deep cortical layer NMDA connections), the ICC reliabilities were excellent (>0.8). The two superficial AMPA connections and a deep NMDA connection had ICC > 0.6, *P* < 0.005. The schematic in Fig. 4D illustrates the reliability of each of the connections.

**Figure 5:**
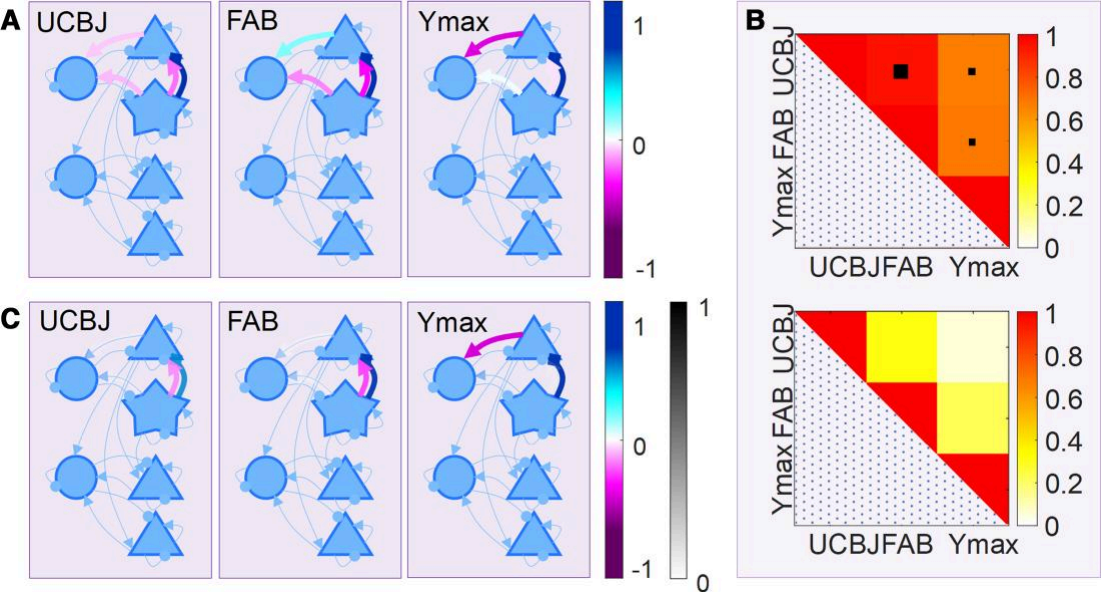
**The relationship between connectivity, synaptic loss, clinical impairment and evoked responses**. (**A**) The connections found to be significantly correlated with ^11^C-UCB-J, FAB scores and the maximal mismatch-window deflection in the event-related field (ERF) (*Y*_max_) are shown as thick arrows, coloured according to correlation magnitude. (**B**) *Top*: The correlation matrix created from parameter values for the influences of ^11^C-UCB-J, FAB and *Y*_max_ as PEB covariates, indicating the similar influences of on the posterior means of connectivity parameters within the cortical microcircuit. The larger black box denotes *P* < 0.001, the smaller black box denotes *P* < 0.05. *Bottom*: The correlation matrix for the three covariates used in the PEB, ^11^C-UCB-J, FAB and *Y*_max_, for which all *P* > 0.05 (NS). (**C**) Connections that are likely to be related to clinical, synaptic and evoked response metrics following Bayesian model reduction, coloured according to correlation magnitude and transparency according to posterior probability (see Supplementary Fig. 3 for parameter values).

We tested the relationship between the each of the superficial and deep cortical AMPA, NMDA and GABA connections and the two independent measures of disease severity: (i) the FAB; and (ii) the maximal deflection in the mismatch response (*Y*_max_). Using empirical priors derived from individuals’ PET, FAB and *Y*_max_ values, PEB yielded similar profiles in terms of the priors’ influence on connectivity (Fig. 5A). For the set of connections illustrated in Fig. 5A, the influences of ^11^C-UCB-J, FAB and *Y*_max_ covariates were highly correlated (Fig. 5B, upper). This high correlation in the influence of the three severity measures was observed despite the low correlation over subjects among the actual ^11^C-UCB-J, FAB and *Y*_max_ measures (Fig. 5B, bottom; see the Supplementary material for details).

Finally, we assessed whether all connections were necessary to explain these effects of synaptic density and measures of disease severity, or whether some connections could be eliminated as redundant (i.e. increasing model complexity more than accuracy). We used Bayesian model reduction to find the best model, after removal of redundant connections. The results are summarized in Fig. 5C: (i) synaptic density, *Y*_max_ and FAB were positively correlated with NMDA activation of superficial pyramidal cells by projections from layer 4 stellate cells; (ii) synaptic density and FAB were negatively correlated with AMPA activation of superficial pyramidal cells by projections from layer 4 stellate cells; and (iii) *Y*_max_ was negatively correlated with AMPA activation of superficial interneurons by projections from superficial pyramidal cells.

## Discussion

There are three principal findings of this study, based on the integration of PET measures of neuropathology with magnetoencephalographic measures of pathophysiology, using PEB-DCM.^35^ First, regional synaptic density had an effect on the functional synaptic gain in a neurotransmitter-specific and laminar-specific fashion. Specifically, the glutamatergic synaptic efficacy of the superficial cortical layer and granular layer of the inferior frontal gyrus, inferred from DCM, was a function of the local synaptic density as measured by ^11^C-UCB-J PET. This corroborates the region- and laminar-specific post-mortem findings in frontotemporal lobar degeneration^16^ where AMPA and NMDA receptors and synaptic density are reduced in the superficial cortical layers of the frontal lobes.^36,37^ Second, we found that even though synaptic density, cognition and the magnitude of the mismatch response were not strongly correlated with each other, the effects of their between-subject variances were mediated by very similar local synaptic gains. Third, the DCM approach was highly reliable in terms of estimating model evidence, which is necessary to test hypotheses through model selection (and model reduction). The full model DCM was highly reliable in terms of the accuracies for the parameters, precision and states. Even at the level of some individual synaptic connections (e.g. AMPA and NMDA), reliability was high despite the posterior dependencies and the multivariate context in which these parameters were estimated.

The relationship between synaptic density and functional change has been examined previously through correlational methods. For example, in Alzheimer’s disease,^6,38^ PSP^10^ and frontotemporal dementia^12^ synaptic density correlates with cognitive function. Magnetoencephalographic evidence of abnormal oscillatory dynamics has been linked to lower ^11^C-UCB-J uptake in the occipital cortex,^7^ while tauopathies have been correlated with spectral differences and spectrally-constrained changes in connectivity in a range of neurodegenerative disorders; including Alzheimer’s disease and frontotemporal dementia.^21,39–43^ These correlative approaches, however, do not directly support inferences on the pathophysiological mechanisms. In preclinical transgenic tauopathy models, it has been possible to study the mechanisms of abnormal neuronal dynamics, confirming the neurophysiological consequences of pyramidal cell depletion and their reduced synaptic density.^44–46^ Despite the presence of tauopathy, these models differ from sporadic human PSP. We chose human PSP as an exemplar condition because of the high clinicopathological correlations and mildness of cortical cell loss, despite marked neurophysiological and cognitive changes associated with the prefrontal cortex.^25,47,48^

PSP impairs cognition, particularly in domains associated with frontal cortex such as executive function, cognitive flexibility, response inhibition, verbal fluency and social cognition.^22,25,49–51^ These deficits are common in PSP Richardson’s syndrome, and are prominent at the presentation of PSP-Frontal phenotype (but not restricted to it). The severity of cognitive change, despite the generally mild cortical atrophy, led to the hypothesis that the impact of PSP on cognition and cognitive physiology is the result of cortical synaptic loss.^10^ By translating the synaptic loss in PSP into a generative model of cortical neurophysiology, one can begin to focus on candidate solutions with targeted pharmacology,^19^ and link to preclinical models of the synaptopathy in genetic tauopathies.^52^ The formal integration of synaptic density into the canonical microcircuit, in which synaptic density forms subject- and cell-specific empirical priors on the microcircuit, goes beyond previous correlative approaches. Given the multivariate nature of the cortical circuits, the use of model comparison—rather than univariate analyses of mean *a posteriori* parameter estimates—properly accommodates the posterior covariance among parameters and increases reliability; two highly desirable properties when anticipating interventional studies.

We probed the cortical circuits using responses evoked in the roving auditory mismatch paradigm. Such tasks and change detection paradigms have been used to study many forms of dementia, ageing and other neurological diseases.^53–56^ This paradigm reliably evokes signals in temporal, parietal and frontal regions.^57^ The relative simplicity of the task and robustness of the activity it generates makes the paradigm highly suited to these types of modelling.^19,29^

Owing to the canonical nature of the local network and the optimization procedure for inversion between the model and MEG (or EEG) data, there is considerable potential for the extension of this method to other disorders, other brain regions and other multi-modal markers of pathology. The pathology-enriched dynamic causal modelling approach could include other subject-specific markers of pathology, such as magnetic resonance spectroscopy estimates of principal neurotransmitters,^48^ or PET ligand markers for the severity of amyloid or Tau burden^58^ or neuroinflammation;^59^ or even post-mortem quantitative pathology.^60^ Other anatomical regions may be more relevant to hypotheses or mechanisms of other diseases, but the DCM method can be applied to other single regions, or a set of regions connected in networks of distributed sources; with the caveat that increasing complexity of the requisite DCMs may reduce reliability of model inversion.

There are limitations to the study. It is based on data from a small cohort, which could raise the question of type II power for frequentist tests. However, our conclusions are not based on frequentist statistics or the rejection of a null hypothesis. Rather, the ‘power’ in the Bayesian approaches used here derives from making the best sense of data, by posing constrained and informed questions in the form of models or hypotheses and comparing the resulting model evidences. The variational free energy (i.e. a lower bound approximation to log model evidence) differences reported previously means that there is sufficient evidence for hypothesis testing in this cohort, even when accounting for the random effects of being a particular subject, implicit in the PEB analyses. This is not a surprise in view of the severity of PSP, and large effect sizes for both synaptic loss and neurophysiological change.^10^ A related issue is the reliability of the DCM approach, which is not guaranteed in this sort of complex system of modelling. However, we provide evidence of excellent reliability, in terms of the inferences based on model selection and high reliability of many individual parameters. In other studies, where reliability is lower, alternative strategies can be used to achieve robust and accurate estimation with computational efficiency.^61^

A second limitation is that diagnosis was based on clinical criteria, without neuropathology in most of our cases. However, the cases were typical of PSP, for which clinicopathological correlations are very high. Note that cognitive changes are common in those with Richardson’s syndrome and are not confined to those with the PSP-Frontal phenotype.^62^ Third, there is the potential for off-target binding with PET ligands. However, ^11^C-UCB-J has been shown to be reliable and highly correlated with other synaptic markers such as synaptophysin,^63^ and none of the participants were taking a drug treatment known to interfere with the binding of ^11^C-UCB-J (such as levetiracetam). There are also limitations of the neuronal model. We used canonical microcircuit models, extended in accordance with the favourable model evidence in Adams *et al*.^29^ However, these neural mass models are approximations and aggregate many cells and cell types within the broad categories set out. Other connections and neuromodulators may exist, for the task and brain regions concerned. Despite their simplification, models are useful to characterize features of a system under investigation. Our biophysically informed and constrained model aims to recapitulate the neuronal dynamics of the mismatch negativity paradigm, with statistical economy, but we recognize that hypotheses related to other hidden dynamics may call for modification of the model, or analysis of other cortical regions. The PEB approach has the advantage to study the effects of individual differences because the second-level (between-subject) analyses retains the relative uncertainty (i.e. posterior covariance) about parameter estimates, in contrast to frequentist analysis of their expected values. A consequence however is that one could not merely interpolate over the parameters to estimate the connectivity profile of a new subject. As a result, the PEB method is well-suited to interrogate the mechanisms of disease (or treatment), but not to classify new participants from a new single-subject’s model estimation.^64–66^ Other approaches to classification from DCM’s can be taken, such as generative embedding and support vector machines, if this is the researcher’s agenda.^64,66^

In summary, the current study highlights the potential of pathologically enriched DCM to elucidate the mechanisms of human neurodegenerative disease. The methodology is suitable for use with other biomarkers of pathology, from PET or spectroscopy, and the assessment of selective pharmacological interventions that target the mechanisms installed in the model. As a potential platform for experimental medicine, the methodology shows good reliability within session, but future assessment of reliability between sessions and during longitudinal follow-up will be useful. We suggest that this methodology could assist the assessment of novel therapies emerging from preclinical disease models, aiming to preserve or restore human cognitive neurophysiology.

## Supplementary Material

awac471_Supplementary_Data
