# Supplementary material for: Neurophysiological consequences of synapse loss in progressive supranuclear palsy
Source: Brain. 2022 Dec 14;146(6):2584–94. doi: 10.1093/brain/awac471 (PMC10232290; doi:10.1093/brain/awac471)
Supplement: awac471_Supplementary_Data [file awac471_supplementary_data.pdf]

Supplementary Table 1. Demographics and test scores.
Averaged demographic and cognitive data for participants. The mean is shown followed by the standard deviation in brackets.

Supplementary Table 2. Initial model parameters.
The initial mean values of notable model parameters and their variance.

Supplementary Table 3. Subject specific model parameters.
The effect of [11C]UCB-J, FAB and Ymax on connections in the CMC network as summarised in figure 5A and C, in terms of mean parameter values before and after Bayesian Model Averaging, and the posterior probability after Bayesian Model Averaging.

Supplementary Table 1: Demographics and test scores

M:F	6:5
Age	67.5 (10.2)
Education (years)	11.2 (1.5)
ACE-R Total (max=100)	85.6 (6.5)
MMSE (max=30)	28.1 (1.4)
Attention (max=18)	17.6 (0.5)
Memory (max=26)	22.5 (3.6)
Fluency (max=14)	6.5 (3.0)
Language (max=26)	24.2 (1.3)
Visuospatial (max=16)	14.8 (1.2)
FAB (max=18)	14.1 (2.5)
INECO Total (max=30)	19.9 (2.5)
Graded Naming: # correct	19.8 (3.8)
Graded Naming: # errors	10.2 (3.8)
PSPRS (max=100)	24.6 (10.0)
Hayling Total scaled scores	14.8 (3.4)
Hayling overall scaled score	4.4 (1.7)
CBI Total (max=180)	40.4 (25.7)
FRS Percentage	55.1 (29.1)

Supplementary Table 2: Initial model parameters.

Parameter grouping	Specific parameter	Default value	Prior Variance
Hyperparameter	Precision of the noise	4	1/100
Cell influence on dipole	Stellate (J)	0.2	1/45
	Superficial pyramidal (J)	0.8	1/45
	Superficial interneuron(J)	0	1/80
	Deep pyramidal (J)	0.2	1/45
	Deep interneuron (J)	0	1/80
	Thalamic projection (J)	0.2	1/45
	Leadfields	1	1/45
Network connection gains	Intrinsic gains	1	1/8
	Extrinsic gains	1	1/8
Decay constants, τ (ms)	AMPA τ	4	0
	NMDA τ	100	0
	GABAA τ	16	0
	GABAB τ	200	0
	M-current τ	160	0
	H-current τ	100	0
Miscellaneous strengths	K + leak G	1	0
	Background V	2.17	0
Reversal potentials (mV)	Na2 + reversal	60	0
	Ca2 + reversal	10	0
	Cl reversal	−90	0
	K + reversal	−70	0
	IH reversal	−100	0
Firing threshold (mV)	VT (all pops)	−40	0
Firing precision	VX (all pops)	1	0
IHI-V slope	VHX	300	0
Cell Capacitances (pF)	Stellate (C)	200	0
	Superficial pyramidal (C)	150	0
	Superficial interneuron(C)	50	0
	Deep pyramidal (C)	400	0
	Deep interneuron (C)	50	0
	Thalamic projection (C)	200	0
Delays (ms)	Intrinsic	2	0
	Extrinsic cortico-cortical	16	0
	Extrinsic thalamo-cortical	80	0

Supplementary Table 3: Subject specific model parameters.

UCBJ				
Connection		*Mean (pre-BMA)*	*mean (post-BMA)*	*Probability (post-BMA)*
AMPA	ss -> sp	-0.2	-0.16	0.88
AMPA	ss -> si	-0.1	0	0.2
AMPA	sp -> si	-0.08	0	0.2
NMDA	ss -> sp	0.73	0.59	0.86

FAB				
Connection		*Mean (pre-BMA)*	*mean (post-BMA)*	*Probability (post-BMA)*
AMPA	ss -> sp	-0.5	-0.34	0.99
AMPA	ss -> si	-0.22	0	0.31
AMPA	sp -> si	0.24	0	0.37
NMDA	ss -> sp	1.35	1.48	0.92

Ymax				
Connection		*Mean (pre-BMA)*	*mean (post-BMA)*	*Probability (post-BMA)*
AMPA	ss -> sp	-0.04	0	0.12
AMPA	ss -> si	0.02	0	0.13
AMPA	sp -> si	-0.44	-0.46	1
NMDA	ss -> sp	0.88	0.86	0.96
